# Actin Inhibition Increases Megakaryocyte Proplatelet Formation through an Apoptosis-Dependent Mechanism

**DOI:** 10.1371/journal.pone.0125057

**Published:** 2015-04-14

**Authors:** Mauro P. Avanzi, Marina Izak, Oluwasijibomi E. Oluwadara, William Beau Mitchell

**Affiliations:** Platelet Biology Laboratory, New York Blood Center, New York, New York, United States of America; Indian Institute of Technology, INDIA

## Abstract

**Background:**

Megakaryocytes assemble and release platelets through the extension of proplatelet processes, which are cytoplasmic extensions that extrude from the megakaryocyte and form platelets at their tips. Proplatelet formation and platelet release are complex processes that require a combination of structural rearrangements. While the signals that trigger the initiation of proplatelet formation process are not completely understood, it has been shown that inhibition of cytoskeletal signaling in mature megakaryocytes induces proplatelet formation. Megakaryocyte apoptosis may also be involved in initiation of proplatelet extension, although this is controversial. This study inquires whether the proplatelet production induced by cytoskeletal signaling inhibition is dependent on activation of apoptosis.

**Methods:**

Megakaryocytes derived from human umbilical cord blood CD34+ cells were treated with the actin polymerization inhibitor latrunculin and their ploidy and proplatelet formation were quantitated. Apoptosis activation was analyzed by flow cytometry and luminescence assays. Caspase activity was inhibited by two compounds, ZVAD and QVD. Expression levels of pro-survival and pro-apoptosis genes were measured by quantitative RT-PCR. Protein levels of Bcl-XL, Bax and Bak were measured by western blot. Cell ultrastructure was analyzed by electron microscopy.

**Results:**

Actin inhibition resulted in increased ploidy and increased proplatelet formation in cultured umbilical cord blood-derived megakaryocytes. Actin inhibition activated apoptosis in the cultured cells. The effects of actin inhibition on proplatelet formation were blocked by caspase inhibition. Increased expression of both pro-apoptotic and pro-survival genes was observed. Pro-survival protein (Bcl-x_L_) levels were increased compared to levels of pro-apoptotic proteins Bak and Bax. Despite apoptosis being activated, the megakaryocytes underwent minimal ultrastructural changes during actin inhibition.

**Conclusions:**

We report a correlation between increased proplatelet formation and activation of apoptosis, and that the increase in proplatelet formation in response to actin inhibition is caspase dependent. These findings support a role for apoptosis in proplatelet formation in this model.

## Introduction

Thrombocytopenia, or low platelets, is an important medical problem that may result from either platelet destruction or decreased production. Greater understanding of the mechanisms underlying normal platelet production could lead to novel therapies to treat thrombocytopenia. Platelets are produced by megakaryocytes in the bone marrow. Megakaryocytes assemble and release platelets through the extension of proplatelet processes, which are cytoplasmic extensions that extrude from the megakaryocyte and traffic granules and organelles to the nascent platelets at their tips [1]. Proplatelet formation and platelet release are complex processes that require a combination of structural rearrangements [2]. While the signals that trigger the initiation of proplatelet formation process are not completely understood, it has been shown that inhibition of cytoskeletal signaling in mature megakaryocytes induces proplatelet formation [3,4]. Megakaryocyte apoptosis may also be involved in initiation of proplatelet extension, although this is controversial [5,6,7,8,9]. While proplatelet formation appears to be caspase-dependent, platelet production in mice was apparently normal in the absence of the apoptosis pathway.

The mechanisms of apoptosis involve two parallel cascades of molecular events, the intrinsic and the extrinsic pathways [10,11]. The intrinsic, or mitochondrial pathway, involves a diverse array of non-receptor mediated stimuli that produce intracellular signals that act directly on the mitochondria. These stimuli cause changes in the inner mitochondrial membrane and result in membrane potential loss, followed by mitochondrial release of cytochrome c [12,13]. Cytochrome c release then results in activation of downstream caspases. The control of these mitochondrial events is mainly regulated by members of the Bcl-2 family proteins. Bcl-2 members are either pro-survival proteins (BCL-2, BCL-XL, BCL-x) or pro-apoptotic proteins (BAK, BAX, BID, BIM) and the fine-tuned balance between these members determines whether apoptosis is activated [10,14]. The extrinsic pathway involves transmembrane receptor-mediated interactions, including Fas-ligand and TNF-α, that activate downstream procaspase-8 and consequently caspase-8. Once caspase-8 is activated the execution phase of apoptosis is triggered [10]. Both pathways ultimately converge on the execution pathway of activated caspases, especially effector caspase-3 and caspase-7 [15]. This process induces typical cellular morphologic changes, such as cytoskeleton reorganization and nuclear shrinking [16].

Members of the BCL-2 family have been shown to be directly involved in megakaryocyte maturation and apoptosis [17,18,19]. There is morphological evidence of a connection between apoptosis and the process of proplatelet formation in that late stage megakaryocytes exhibit several visual hallmarks of apoptosis [20]. Initiation of proplatelet formation was reported to be a caspase-dependent process [5,9,18], and caspase activation lead to increased proplatelet formation [21]. In addition, stimulation of megakaryocyte apoptosis with nitric oxide increased proplatelet formation in culture [22]. Several studies, however, have questioned the requirement of apoptosis for proplatelet formation. Caspases were reported to be dispensable for megakaryocytes to generate platelets [23]. The pro-apoptotic Bak and Bax were also shown to be dispensable for normal platelet formation [6]. However, the intrinsic pro-survival pathway was required for megakaryocytes to adequately form proplatelets and release platelets [6,24]. Thus, the role of apoptosis in megakaryocyte proplatelet formation is still not clear.

Since inhibitors of cytoskeleton protein signaling, particularly actin polymerization inhibitors, are also known to induce apoptosis in cancer models [25,26], we examined whether the actin polymerization inhibitor latrunculin induced proplatelet formation through an apoptosis-dependent mechanism. We analyzed the expression of pro-survival and pro-apoptotic mRNA, proteins and cellular ultra-structure. We report a correlation between increased proplatelet formation and activation of apoptosis, and that the increase in proplatelet formation is caspase dependent. We found upregulation of both pro-apoptotic and pro-survival genes occurs during this process. Interestingly, we also found that pro-survival protein (Bcl-x_L_) levels are increased compared to pro-apoptotic proteins (Bak, Bax), further supporting a critical role for Bcl-xL in proplatelet formation.

## Material and Methods

### Cell culture

Megakaryocytes were derived from umbilical cord blood stem cells, as previously described [3]. Units of umbilical cord blood unsuitable for clinical use were provided by the National Cord Blood Program at the New York Blood Center, http://www.nationalcordbloodprogram.org. Use of umbilical cord blood units was approved by the Institutional Review Board of New York Blood Center. Briefly, umbilical cord blood-derived CD34+ cells were enriched by a negative selection method with RosetteSep (Stem Cell Technologies, Vancouver, Canada). Cells were plated in 24 well plates at 1 x 10^5^ cells (TNC)/ml of Stemspan SFEM medium (Stem Cell Technologies) with 50ng/ml thrombopoietin (TPO) (Millipore, Temecula, CA) and 5ng/ml Stem Cells Factor (SCF) (Millipore). For some assays megakaryocytes were enriched on day 8 or day 12 by selection with anti-CD61 magnetic beads (Miltenyi Biotech, Auburn, CA) and cultured with TPO 50ng/ml until analysis.

### Induction of polyploidization

Day 8 megakaryocytes that had been positively selected for CD61 expression were stimulated to undergo polyploidization by adding an actin polymerization inhibitor (Latrunculin, Santa Cruz Biotechnology, Dallas, TX, USA) in a final concentration of 10μM. Untreated cells were used as control. In order to investigate the effect of apoptosis inhibition in megakaryocytes two different anti-apoptosis agents were tested. ZVAD-fmk (Promega, Madison, WI, USA) in a concentration of 20μM and Q-VD-OPh (MP Biomedicals, Solon, OH, USA) in concentration of 25μM were separately added on day 8 of some experiments. Some megakaryocytes were treated with a Myosin Light Chain kinase inhibitor (MLCKI) on day 8 in a concentration of 50μM as a control for inhibition of cytoskeleton function.

### Morphologic Analysis

Cells collected on day 11 were cytospun onto glass slides using a Cytospin 4 centrifuge (Thermo Scientific, Springfield, NJ), stained with Wright-Giemsa (Fisher Scientific, New York, NY), and observed with a Leica inverted contrasting microscope fitted with a camera (DFC420, Leica Camera, Inc, Allendale, NJ)

### Fluorescence microscopy

On day 11 of culture megakaryocytes were analyzed by fluorescence microscopy. Megakaryocytes were attached to Poly-L-Lysine coated slides, fixed and permeabilized. Polyploid megakaryocyte analysis was performed using anti-GPIIb3a antibody (generous gift from Dr. Barry Coller, Rockefeller University) conjugated with Alexa Fluor 647. Proplatelet formation analysis was performed using anti-beta-1-tubulin rabbit anti-human polyclonal antibody (generous gift from Dr. Joseph Italiano Jr, Harvard University) and goat anti-rabbit Alexa Fluor 514 secondary antibody (Life Technologies, Carlsbad, CA). Megakaryocytes were analyzed with a Leitz Aristoplan fluorescence microscope (Leica, Allendale, NJ) fitted with an Olympus C8484-51 camera (Olympus, Center Valley, PA).

### Ploidy analysis

Megakaryocyte ploidy was analyzed on day 11 of culture. Cells were labeled with APC-conjugated anti-CD41 antibody, incubated for 20 minutes on ice, fixed with 2% paraformaldehyde (Fischer Scientific) for 15 minutes and then washed with PBS (Cellgro, Manassas, VA). Next, cells were permeabilized with a BSA 0.02% + saponin 0.005% (MP Biomedicals, Solon, OH, USA) and incubated for 15 minutes at room temperature. Finally cells were treated with Propidium Iodide (PI)/RNase solution (BD, Franklin Lakes, NJ) and incubated for 30 minutes in dark at room temperature before flow cytometry analysis.

### Proplatelet formation quantification

Megakaryocytes and proplatelet formations were counted and analyzed on day 11 of culture using a grid strategy, as previously described [3]. Briefly, using an inverted light microscope (Olympus CKX41) 10 random-field images were acquired at 40x magnification from each well. All megakaryocytes present on each field were considered for counting. A counting grid with 25μm squares was used to enumerate the number of proplatelet processes. Each proplatelet segment inside each square was counted and the sum of all segments in 10 random field images (40x magnification) was used for comparison. From these numbers, a measure of proplatelet density per cell per unit area was generated and used to compare the results of different culture conditions.

### Apoptosis analysis

Apoptosis activation was analyzed and quantified in mature megakaryocytes by measuring Mitochondrial Outer Membrane Potential (MOMP) and phosphatidyl serine (PS) externalization using flow cytometry with the Mitochondrial Membrane Potential Apoptosis Kit with Mitotracker Red and Annexin V Alexa Fluor 488 (Molecular Probes, Life Technologies) according to manufacturer’s instructions. Megakaryocytes were considered apoptotic if they had high expression of both MOMP (positive Mitotracker using PE Texas Red staining) and PS (positive Annexin V staining, FITC). Caspase 3 and 7 activity were measured using Caspase-Glo 3/7 Assay (Promega, Madison, WI), according to manufacturer’s instructions.

### Electron microscopy analysis

On day 11 of culture megakaryocytes treated with latrunculin and control cells were fixed with 2.5% Glutaraldehyde (Electron Microscopy Sciences, Hatfield, PA) and prepared for electron microscopy analysis. Ultrathin sections were sectioned on RMC MTX ultramicrotome (Boeckeler Instruments, Tucson, AZ) and imaged on an FEI Tecnai 12 spirit transmission electron microscope (FEI, Morristown, NJ) with an AMT camera (Advanced Microscopy Techniques, Woburn, MA).

### Quantitative (Q)-RT-PCR

Quantitative reverse transcription polymerase chain reaction (Q-RTPCR) was performed on RNA from day 11 megakaryocytes (both treated and untreated) using Power SYBR Green RNA-to-CT (Applied Biosystems, Grand Island, NY). The following primers were used: BAK, BAX, BNIP3, BNIP3L, BCL2, BCL-XL, IGF1R, CFLAR (all Qiagen, Germantown, MD). PCR reactions were performed on a Viia7 cycler (Applied Biosystems). The amount of mRNA for each sample was normalized using beta-actin as endogenous control.

### Protein analysis

On day 11 of culture proteins were extracted from megakaryocytes, quantified, boiled for 10 min at 95°C and subjected to SDS gel electrophoresis (Mini-Protean II System; Bio-Rad Laboratories, Richmond, CA). Equal amounts (70μg) of protein were loaded into each well. The separated proteins were then transferred to nitrocellulose membrane. After transfer, the nitrocellulose membranes were blocked with blotting-grade blocker 5% (Bio-Rad) for 1 h. Membranes were reacted with primary rabbit anti-human antibodies against BAK, BAX or BCL-2 (Cell Signaling Technology, Danvers, MA, USA) diluted 1:100 in blocking buffer, and then incubated overnight in a rocker at 4°C. The membranes were washed with 25mM Tris-buffered saline containing 0.005% Tween-20 (TTBS), and then reacted with a secondary goat anti-rabbit antibody conjugated with horseradish peroxidase (Cell Signaling Technology) at a dilution of 1:1,000 in TTBS for 2 h at room temperature. The membranes were then washed in TTBS and developed using SuperSignal West Pico chemiluminescent substrate (Thermo Scientific) according to the recommendations of the manufacturer, exposed to film (Thermo Scientific), and developed using a Konica SRX-101A (Konica Minolta, Newark, NJ, USA) developer. The films were imaged using UVP EC3 Imaging System (UVP, Upland, CA, USA) and the band density was quantified using UVP Vision Works LS program (UVP). All protein profiles were compared to actin controls: primary monoclonal mouse anti-human actin antibody (Sigma-Aldrich) was reacted at 1:20,000 dilution for 1 hour and secondary ECL anti-mouse IgG conjugated to horseradish peroxidase was reacted at 1:3,000 dilution for 2 hours.

### Intracellular protein analysis by flow cytometry

Intracellular proteins Bcl-xl and Bim were quantified in latrunculin-treated and control megakaryocytes using a flow cytometry assay. Briefly, day 11 megakaryocytes were incubated with APC-conjugated anti-CD41 antibody for 20 minutes at 37°C, fixed with 1% Paraformaldehyde (Fischer Scientific) for 15 minutes and then washed with PBS (Cellgro, Manassas, VA). Cells were then permeabilized with a BSA 0.02% + saponin 0.005% (MP Biomedicals, Solon, OH, USA) solution for 15 minutes at room temperature, and then incubated with rabbit anti-human antibody Alexa-488-conjugated anti-BCL-xl and PE-conjugated anti-BIM antibodies (Cell Signaling Technology) at a 1:50 concentration. Cells were then washed with PBS and analyzed with flow cytometry.

## Results

### Polyploidization and proplatelet formation induced by latrunculin are caspase dependent

**Fig 1:**
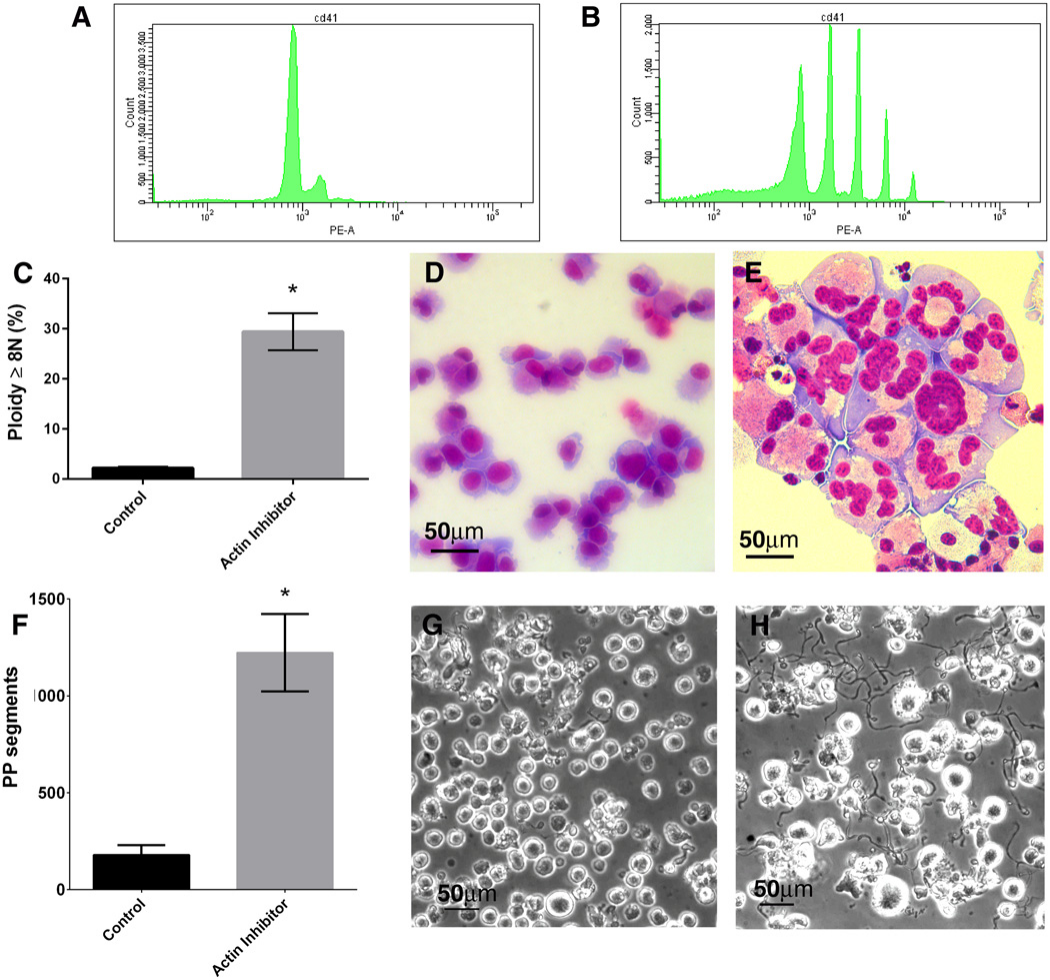
Actin polymerization inhibitor (Latrunculin) increases megakaryocyte maturation and proplatelet formation. A,B) FACS profiles of propidium iodide staining indicating ploidy of control (A) and latrunculin treated (B) cells. C) Actin inhibition increases the levels of polyploid megakaryocytes in culture. Y axis represents the % of cells with ploidy ≥8N. D,E) Wright-Giemsa stained megakaryocytes showing increased polyploidization with actin-inhibitor treatement: D) Untreated control E) Actin polymerization inhibitor treated. F) Actin inhibition increases the levels of megakaryocytes proplatelet formation in culture. Y-axis represents the sum of all proplatelet (PP) segments counted inside of grids in 10 random field images (40x magnification). G,H) Light microscopy images showing increased proplatelet formation after actin polymerization inhibitor treatment: G) Untreated control H) Actin polymerization inhibitor treated. * p<0.05

**Fig 2:**
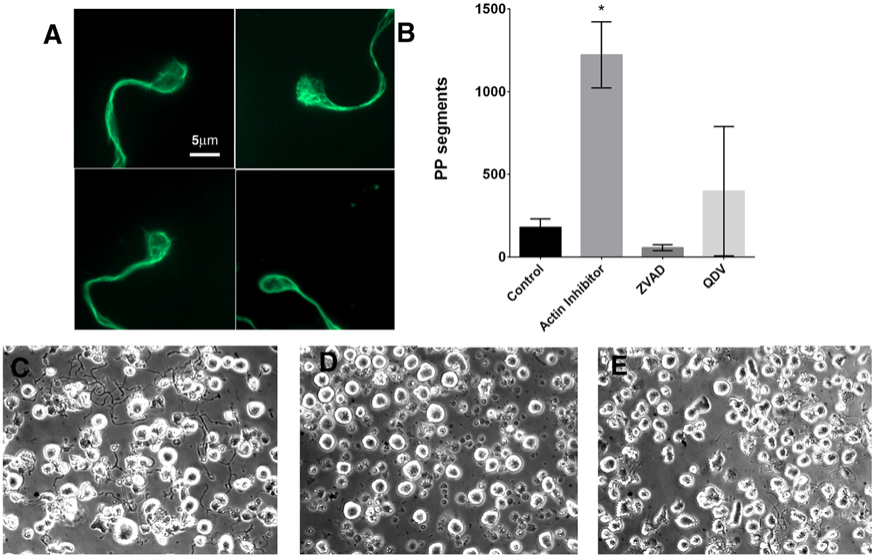
Actin polymerization inhibitor (Latrunculin) treatment does not affect proplatelet formation and platelet structure. A) Preplatelet-like cell protrusions stained with β- tubulin and showing characteristic circumferential loops of microtubules. B) Caspase inhibition with ZVAD-FMK or Q-VD-OPH inhibits proplatelet formation from actin polymerization inhibitor treated megakaryocytes. Y-axis represents the sum of all proplatelet (PP) segments counted inside of grids in 10 random field images (40x magnification). C,D) Light microscopy images showing decreased proplatelet formation after caspase inhibitor treatment: C) Actin polymerization inhibitor treated D) ZVAD-FMK E) Q-VD-OPH. * p<0.05

Polyploidization was induced in day 8 megakaryocytes that had been positively selected for CD61 expression. Treatment with the actin polymerization inhibitor Latrunculin significantly increased the number of megakaryocytes that reached a ploidy ≥8N compared to untreated controls (p = 0.03) (Fig 1A–1E). Treatment with actin polymerization inhibitor also increased the number of proplatelet formations released by each megakaryocyte when compared to untreated controls (p = 0.02) (Fig 1F–1H). Proplatelet microtubule structure was analyzed for β-tubulin with fluorescent microscopy, revealing characteristic circumferential microtubule coils at the end of each proplatelet (Fig 2A). Proplatelets were similar in dimension to previously reported proplatelets[27]. This indicated that in our culture system, a single exposure to actin polymerization inhibitor on day 8 was compatible with development of grossly normal proplatelet cytoskeleton structure. To determine whether apoptosis pathways were involved in this process of increased proplatelet formation, we inhibited caspase activation with two different caspase inhibitors, ZVAD-fmk and Q-VD-OPh. While zVAD-fmk has a range of non-caspase targets the latter is a more specific caspase inhibitor. Proplatelet formation was inhibited in latrunculin-treated cells treated with both ZVAD-fmk and Q-VD-OPh (Fig 2B–2D) (both p<0.05) suggesting that caspase activation was globally involved in the increased proplatelet formation seen with the actin polymerization inhibitor.

### Latrunculin induces megakaryocyte apoptosis

**Fig 3:**
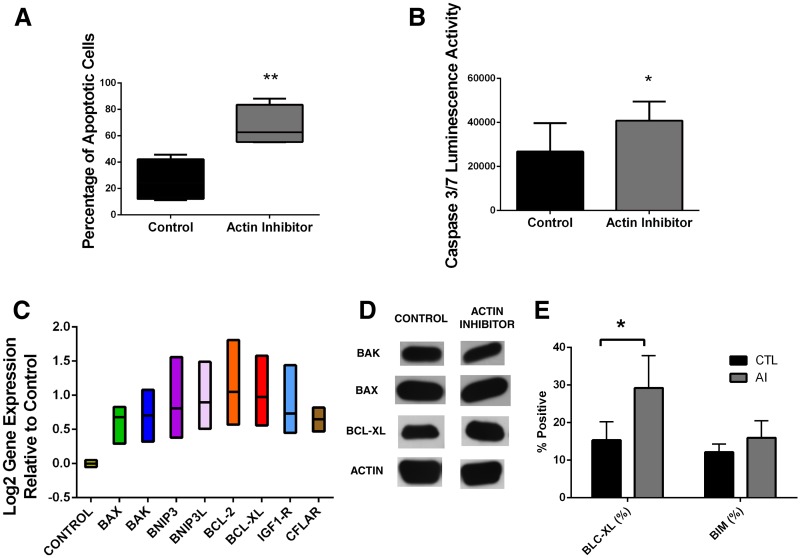
Actin inhibitor (Latrunculin) induces apoptosis activation in cultured megakaryocytes. A) FACS analysis of apoptosis activation by measuring Mitochondrial Outer Membrane Potential (MOMP) and phosphatidyl serine (PS) externalization B) Luminescence activity measuring Caspase 3 and 7 activity were measured using Caspase-Glo 3/7 Assay. Actin inhibition induces overexpression of both pro-apoptotic and pro-survival genes and increases BCL-XL protein synthesis C) RT-PCR analysis of mRNA expression of pro-apoptotic genes: BAX, BAK, BNIP3 and BNIP3L and pro-survival genes: BCL-2, BCL-XL, IGF1R and CFLAR relative to untreated control. BCL-XL protein showed increased synthesis with actin polymerization inhibitor treatment D) Western Blot (density ratio = 1.34 compared to untreated control) and E) Intracellular protein quantification with FACS. * p<0.05; ** p<0.005

Since both ZVAD-fmk and Q-VD-OPh inhibited the proplatelet formation induced by latrunculin, we examined those cells for evidence of apoptosis activation. Apoptosis was analyzed and quantified with flow cytometry by measuring alterations in the Mitochondrial Outer Membrane Potential (MOMP) and the externalization of phosphatidyl serine (PS) in the cell surface. Apoptosis was also measured by analysis of caspase 3 and 7 activation by luminescence. Megakaryocytes treated with actin polymerization inhibitor had a higher percentage of apoptotic cells in both flow cytometry (p = 0.03) and luminescence (p = 0.05) compared to untreated control (Fig 3A and 3B). To determine whether the effect of latrunculin was simply due to toxicity, treated cells were stained with the dead cell-specific stain, 7AAD. The latrunculin treated wells had only slightly higher percent of dead cells than the control wells, 20% +/- 8% vs 35% +/- 4%, respectively, on day 11 of culture. Thus the increase in apoptotic cells is not accounted for only by toxicity.

### Latrunculin increases expression of pro-apoptotic and pro-survival genes

Since apoptosis was activated in the latrunculin-treated cells, we analyzed the expression of genes involved in apoptosis. We analyzed the expression of genes that were related to apoptosis (BAK, BAX, BNIP3, BNIP3L) or to cell survival (BCL2, BCL-XL, IGF1R, CFLAR). Somewhat surprisingly, this analysis showed that both pro-apoptotic and pro-survival genes were upregulated: BAK (p = 0.008), BAX (p = 0.007), BNIP3 (p = 0.01); BNIP3L (p = 0.02); BCL2 (p = 0.04), BCL-XL (p = 0.03), IGF1R (p = 0.01) and CFLAR (p = 0.003) were all upregulated compared to untreated control (Fig 3C). To test whether this effect on apoptosis gene expression was a non-specific result of inhibiting a cytoskeletal protein, we also treated day 8 megakaryocytes with myosin light chain kinase (MLCK) inhibitor, which should target the myosin signaling pathway. Megakaryocytes treated with MLCK inhibitor showed only a modest increase in ploidy and proplatelet formation [3], and had decreased expression of all genes except for BNIP3, which had no difference in expression (data not shown). Thus the effect on both proplatelet formation and apoptosis does not appear to be due to non-specific interference with cytoskeletal signaling.

### Latrunculin causes increased BclxL protein expression

Proteins were extracted from treated and untreated megakaryocytes on day 11 of culture and Bak (25kDa), Bax (20kDa) and Bcl-xl (30kDa) were analyzed by western blot. Protein amounts were quantified by density measurements normalized to an actin control. Comparing the density ratio between actin polymerization inhibitor treated cells and untreated control showed a decrease in Bak protein expression (density ratio = 0.83 compared to control); no change in Bax (density ratio = 1.01) and increase in Bcl-xL protein expression (density ratio = 1.34) (Fig 3D). This finding indicated that even though apoptosis was activated, Bcl-xL protein levels were increased relative to Bak and Bax. Intracellular proteins Bcl-xl and Bim were also quantified using FACS on day 11 of culture. Results showed that Bcl-xl had an increased protein quantification when compared to untreated control (p = 0.003) but not Bim.

### Latrunculin induces only moderate ultrastructural changes

**Fig 4:**
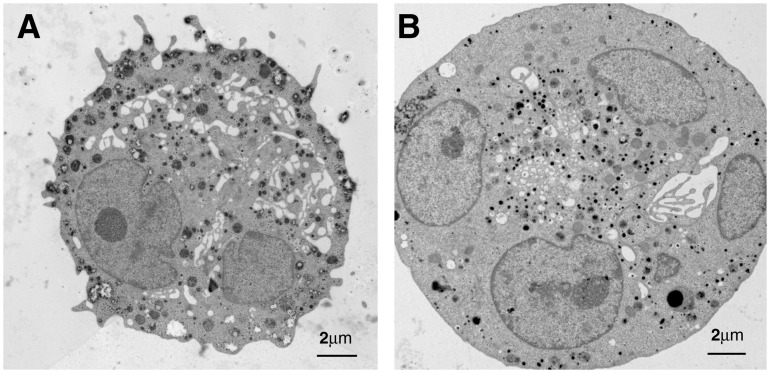
Megakaryocyte ultrastructure showing early signs of apoptosis in actin polymerization inhibitor treated megakaryocytes, such as enlarged peripheral margin with concentration of granules towards the center of the cell and more frequently displayed cytoplasmic blebbing. A) Untreated control B) Actin polymerization inhibitor treated.

The ultrastructural effects on apoptosis of actin polymerization inhibitor treated megakaryocytes were analyzed with transmission electron microscopy and compared to untreated control. Control and latrunculin treated megakaryocytes both showed normal presence of nuclei, granules and mitochondria (Fig 4). However, latrunculin treated megakaryocytes showed enlarged peripheral margin with concentration of granules towards the center of the cell and more frequently displayed cytoplasmic blebbing (Fig 4). These characteristics are similar to early signs of apoptosis. Control cells, on the contrary, showed normal granule distribution and no cytoplasmic blebbing (Fig 4). No further ultrastructural evidence of apoptosis was found.

## Discussion

The presented results further develop the relationship between apoptosis and proplatelet formation, indicating that both pro-apoptotic and pro-survival pathways play key roles in triggering platelet formation. The actin polymerization inhibitor latrunculin increased megakaryocyte polyploidization, increased the amount of proplatelet formation, and is a known inducer of apoptosis [25]. Mature megakaryocytes treated with latrunculin released more proplatelets and also had an overall increased apoptosis activation profile. Interestingly, these cells displayed an increased expression of both pro-apoptotic (BAK, BAX, BNIP3, BNIP3L) and pro-survival genes (BCL-2, BCL-XL, IGF1R and CFLAR). Expression of these genes is normally increased in both megakaryocyte development and apoptosis [28]. These findings support the idea that proplatelet formation is triggered by activation of both pro-apoptotic and pro-survival pathways. Our finding that Bcl-xL is increased after latrunculin treatment supports the idea that Bcl-xL must be active throughout proplatelet formation, keeping the megakaryocyte alive despite activation of apoptosis pathways. This is in line with the finding that Bcl-xL was necessary for platelet formation [6]. The ability of latrunculin to induce proplatelet formation was caspase-dependent, since it was decreased by both ZVAD-FMK and Q-VD-OPh. In contrast to the latrunculin-treated cells, megakaryocytes treated with MLCKI showed down-regulation of both pro-apoptotic and pro-survival genes. Additionally megakaryocytes treated with MLCKI also had lower levels of polyploidization and extended fewer proplatelets. Together these findings suggest that it was the induction of apoptosis that induced proplatelet formation in the latrunculin-treated cells, and not just the overall inhibition of cytoskeleton function.

Several factors augment the process of proplatelet formation, including polyploidization, interaction with extra cellular matrices proteins, and shear stress, [3,29,30]. However, a consensus has not been reached on the idea that megakaryocytes undergo apoptosis to release proplatelets [8,9,24]. The apoptosis hypothesis was based upon data showing that late stage human megakaryocytes presented typical apoptosis ultrastructural morphology, overexpressed pro-apoptotic proteins and displayed activated caspase signaling throughout the proplatelet formation process [5,31]. In addition, it was shown that the pro-apoptotic stimulus nitric oxide could trigger the release of platelet-like particles from megakaryocyte cell line Meg-01 [22,32]. Recently, however, it has been demonstrated in mice that thrombopoiesis was unperturbed in the absence of caspase-9 and that the apoptotic caspase cascade was not required for platelet production [23,33]. Additionally, loss of BAK and BAX had no impact upon platelet production in a mouse model, suggesting that the intrinsic pathway was not required for platelet formation [8]. However, the pro-survival protein Bcl-xL was required for megakaryocytes to survive until platelet formation, indicating that the intrinsic apoptosis pathway was activated at least during the last stages of megakaryocyte maturation and early platelet formation [6]. Interestingly, overexpression of Bcl-xL reduced platelet formation, suggesting that quelling intrinsic pathway was detrimental to proplatelet formation [18]. These data and the findings of the current study suggest that a well-tuned level of Bcl-xL is required to counter the intrinsic pathway activation occurring during the last stages of megakaryocyte maturation, even though that activation may not be strictly necessary for the process of platelet formation [6,8]. It is also possible that initiation of proplatelet formation in our model, which is based on disruption of the actin cytoskeleton in cultured human CD34+ cells, may differ mechanistically from the mouse knock-out model.

Bcl-xL protein expression was increased in latrunculin-treated megakaryocytes in concurrence with upregulation of Bcl-xL mRNA. While Bcl-xL is a known regulator of apoptosis in megakaryocytes, its expression during proplatelet formation is not clearly defined. Bcl-xL was reported to be up-regulated during megakaryocyte differentiation but absent during final stages of senescence [19]. However, Bcl-xL expression was also reported to continuously increase throughout megakaryocyte development and to be post translationally regulated by thrombopoietin-mediated Akt activation [34]. Our finding that Bcl-xL was present during proplatelet formation supports the latter findings. Although overexpression of Bcl-xL in late stage megakaryocytes was shown to impair platelet fragmentation in mice [18], the increased levels of Bcl-xL found in our experiments suggest that moderate up-regulation may be beneficial to the process of proplatelet formation. In contrast to Bcl-xL, protein levels of the pro-apoptotic proteins, Bak and Bax, were not increased despite upregulation of their genes. Both pro-apoptotic proteins Bak and Bax expression are post-translationally regulated, possibly explaining the lack of correlation between gene and protein expression[35].

The current findings indicate that in our system, proplatelet formation induced by latrunculin is dependent on apoptosis. RNA and protein analysis indicated that both the pro-survival factors and pro-apoptotic factors may play a role in this process. Proplatelet formation could also have been impacted by other members of the intrinsic pathway, such as BIM and BID, which can activate apoptosis independently of BAK and BAX [36,37]: these could be the focus of future studies. Another possibility is that the apoptosis trigger is through the extrinsic pathway via signaling by TNF-α/INF-gamma and activation of caspase 8 [10]. Finally, the proplatelet formation process could have been induced by an alternative programmed cell death pathway instead of the classical caspase-dependent apoptosis [38]. However, our finding that inhibition of caspase with ZVAD-FMK and Q-VD-OPh decreased proplatelet formation favors a classical caspase-related process.

Our findings provide new information about the functional relationship between apoptosis and proplatelet formation in human cells and demonstrate the importance of a fine-tuned balance between apoptosis and survival factors during this process. Deciphering the role of apoptosis in proplatelet formation will not only shed light on the basic cell biology behind platelet biogenesis but may also enhance our understanding of congenital and acquired thrombocytopenia. For example, decreased megakaryocyte apoptosis has been proposed as a mechanisms underlying immune thrombocytopenia[39]. Greater understanding of apoptosis in platelet production opens possibilities to new therapeutic strategies to treat thrombocytopenic disorders, and to optimize the process of ex vivo expansion of platelets from stem cells.
